# Association between Blood Pressure and HIV Status in Rural Uganda: Results of Cross-Sectional Analysis

**DOI:** 10.5334/gh.858

**Published:** 2021-02-10

**Authors:** Anxious J. Niwaha, Adaeze C. Wosu, Alex Kayongo, Charles Batte, Trishul Siddharthan, Robert Kalyesubula, Bruce Kirenga, William Checkley

**Affiliations:** 1 Non-Communicable Diseases (NCD) Theme, MRC/UVRI and LSHTM Uganda Research Unit, Entebbe, UG; 2 Department of Epidemiology, Johns Hopkins University Bloomberg School of Public Health, Baltimore, Maryland, US; 3 Center for Global Non-Communicable Disease Research and Training, School of Medicine, Johns Hopkins University, Baltimore, Maryland, US; 4 School of Medicine, Makerere University College of Health Sciences, Kampala, UG; 5 Lung Institute, Makerere University College of Health Sciences, Kampala, UG; 6 Division of Pulmonary and Critical Care Medicine, Johns Hopkins University, Baltimore, Maryland, US; 7 African Community Center for Social Sustainability (ACCESS), Nakaseke, UG

**Keywords:** HIV, hypertension, blood pressure, Non-communicable diseases, NCDs

## Abstract

**Introduction:**

The association between HIV status and hypertension is not well described within sub-Saharan Africa. We examined prevalence and risk factors for hypertension among HIV positive and negative individuals living in a rural district of Uganda.

**Methods:**

We conducted a cross-sectional analysis in two concurrent cohorts of 600 HIV negative and 721 HIV seropositive individuals aged ≥35 years.

**Results:**

Of the 721 HIV positive participants, 59.8% were women and the median age was 44.3 years, while for HIV negative individuals, 55% were women and the median age was 47.8 years. Over 90% of HIV positive individuals were on antiretroviral treatment. The prevalence of hypertension (≥140/≥90 mmHg) was 33.5% in HIV negative individuals and 23.9% in HIV positive individuals. Age (adjusted OR = 1.05, 95% CI 1.03 to 1.06) and BMI (adjusted OR = 1.08, 95% CI 1.05 to 1.12) were associated with higher odds of hypertension. Having HIV was associated with lower odds of hypertension (adjusted OR = 0.66, 95% CI 0.50 to 0.88), lower systolic blood pressure (–5.1 mmHg, 95% CI: –7.4 to –2.4) and lower diastolic blood pressure (–4.0 mmHg, 95% CI: –5.6 to –2.5). We did not observe differences in the odds of hypertension by CD4 count, viral load or ART among HIV positive individuals in this sample.

**Conclusions:**

Hypertension was prevalent in one third of HIV negative individuals and in one fourth of HIV positive patients. While access to health information among individuals attending HIV clinics may explain observed differences, more research is needed to understand plausible biological and social mechanisms that could explain lower blood pressure among people living with HIV in Uganda.

## Introduction

People living with HIV (PLHIV) now have an increased life expectancy as a result of successful scale up and sustained access to antiretroviral therapy (ART) in Uganda and other countries in sub-Saharan Africa (SSA). The total number of AIDS-related deaths in Uganda declined from 56,000 in 2010 to 23,000 in 2018 [1]. However, hypertension rates increased among people living with HIV in the same time [2,3]. For example, a recent retrospective study found that the prevalence of hypertension at an urban HIV clinic rose from 16.9% to 32.3% between 2009 and 2013 [3]. Moreover, increasing prevalence of hypertension has been reported in both the general population and among HIV positive individuals in Uganda and other SSA countries [2,4].

Although a number of studies, especially from high income countries, and a recent meta-analysis [5] have demonstrated an increased risk of high blood pressure among HIV positive individuals; other studies from Uganda [6,7] and other SSA countries report lower prevalence of hypertension in HIV positive individuals compared to HIV negative individuals [8]. These paradoxical results reinforce the need for more studies examining associations between HIV and blood pressure and potential mechanisms underlining these associations.

Programs are already underway to integrate non-communicable disease (NCD) management within routine HIV care for PLHIV in Uganda and other SSA countries. Understanding the burden of high blood pressure and factors that modify its risk in the context of HIV, including HIV severity (detectable vs. undetectable viral load), duration and ART among others, will help inform policy and HIV programmers in the integration of hypertension and HIV care services. In this study, we examined the prevalence of hypertension and associated risk factors among HIV positive and negative adults living in a rural district of Uganda.

## Methods

### Study setting

We conducted secondary analysis of pooled data from two cohort studies carried out in Nakaseke district, Central Uganda. Specifically, data were compiled from the community-based Lung function in Nakaseke and Kampala (LiNK) study and the Lung function in HIV positive individuals (HIV-LINK) study, which was conducted at four HIV/AIDS treatment centers in Nakaseke district, Uganda [9].

### Study design

LiNK is a community-based cohort study conducted between November 2015 and June 2016 and has been described elsewhere [9]. HIV-LiNK is a cross-sectional study that recruited HIV positive participants at four HIV/AIDS treatment centers in Nakaseke district, Uganda, between January and December 2017 [10].

### Participants and sampling

**Figure 1:**
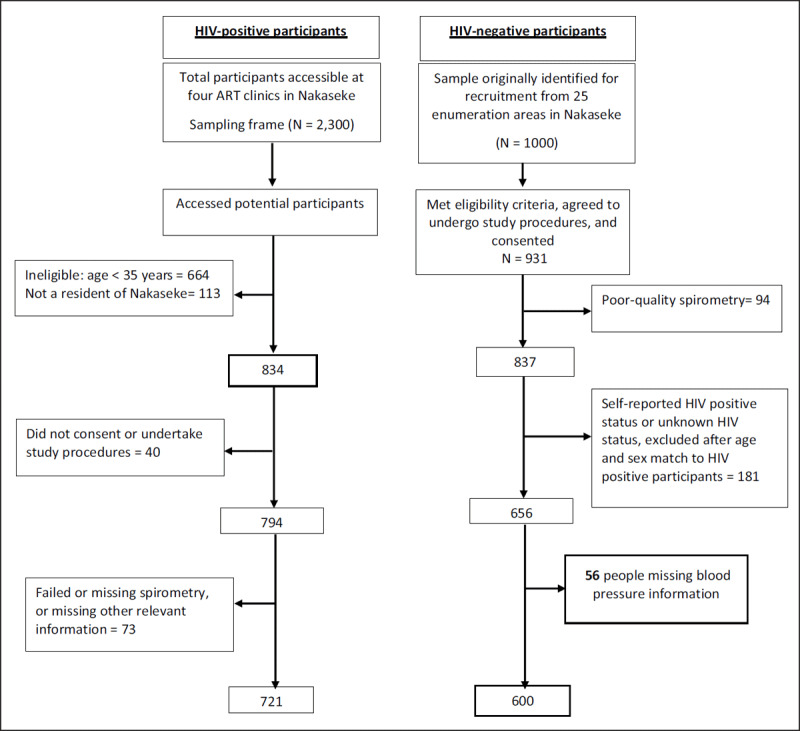
Flow chart for recruitment of HIV-positive and HIV-negative participants and inclusion in the final blood pressure analysis.

The details of recruitment of HIV positive participants have been published elsewhere [9]. Briefly, HIV positive individuals were recruited from centrally located ART clinics that provide comprehensive HIV services to over 13,000 HIV positive individuals within Nakaseke district. Participants were eligible for inclusion if they resided within Nakaseke district and were at least 35 years of age, HIV seropositive, capable of understanding study procedures and provided written informed consent. HIV treatment guidelines at the time of the study recommended initiation of ART for all patients who tested HIV positive irrespective of their CD4+T cell count. Participants with HIV were identified as seropositive on an enzyme-linked immunosorbent assay and/or Western blot according to ART clinic guidelines. Of 2,300 HIV positive individuals registered at ART clinics in Nakaseke, 1,611 potential participants were contacted through regular ART clinic visits, of which 834 (51.8%) met the eligibility criteria for the parent study, and 721 HIV positive participants were maintained in the final analytical sample for the blood pressure (BP) analysis. Please see the participant flow chart (Figure 1) for flow chart of recruitment of HIV positive and HIV negative participants and inclusion in the final blood pressure analysis.

HIV negative participants were recruited from the community as described elsewhere [9]. In brief, 1,000 community participants were randomly selected from 25 enumeration areas in Nakaseke with the probability of selection proportional to the population size of the enumeration area. Fieldworkers conducted home visits between November 2015 and June 2016 to assess eligibility and to obtain informed consent. Inclusion criteria included age ≥35 years, full-time residency in Nakaseke, and capacity to provide informed consent. Exclusion criteria was active pulmonary tuberculosis or a current respiratory infection and pregnancy. Of the 1,000 individuals originally identified for enrolment in LiNK, 931 met inclusion criteria, consented and agreed to undergo spirometry. Ultimately, 837 participants had acceptable and reproducible spirometry results in the parent study. Of these, 181 participants who indicated a positive HIV status or unknown HIV status or could not be age-matched to HIV positive participants were excluded in the final analysis. A further 56 individuals were excluded due to missing BP data. Ultimately, 600 HIV negative participants remained in the final blood pressure analytic sample (see Figure 1). In supplementary file 1: Appendix Table 1, we have included a table describing differences among individuals in our final analytic sample and individuals who were eligible in the parent study.

### Study procedures and outcome variables

Data from each individual encounter in the parent studies were obtained. Information obtained using a questionnaire included socio-demographic, behavioral and clinical characteristics. HIV- related medical history, including CD4+T cell count, viral load, antiretroviral drug use and any reported history of opportunistic infections, was obtained from patient medical records captured in the ART clinics using an electronic medical record system. Current smoking status was defined according to self-reported smoking of tobacco products (daily, occasional, non-smoker). Height and weight measurements were taken in triplicate. BP was measured during household visits for HIV negative participants and at the HIV outpatient clinic for HIV positive participants using a validated semiautomatic oscillometric device (Omron M4; Omron Co. Ltd, Japan) with appropriate cuff size according to arm circumference. Trained nurses performed two consecutive BP measurements two minutes apart after patients rested for at least five minutes in the sitting position with the back and arm supported. The average of the BP readings was used for analysis. For the current analysis, HIV status was the primary exposure of interest while the outcomes of interest were systolic (SBP) and diastolic blood pressure (DBP), as well as hypertension defined as SBP ≥140mmHg or DBP ≥90mmHg.

### Biostatistical methods

First, we assessed distributions of baseline socio-demographic, behavioral and clinical characteristics among HIV positive and HIV negative individuals. Using a definition of hypertension as systolic blood pressure ≥140 mmHg or diastolic blood pressure ≥90mmHg, we computed the proportion with hypertension by HIV status and compared the difference using a chi-square test. To evaluate the independent association of HIV status with the binary hypertension variable, we fitted logistic regression models. Covariates adjusted for include sex, age, marital status, household size, biomass use (charcoal and other fuel sources vs. firewood), highest level of education, body mass index (BMI) and current smoking status.

We further examined the robustness of the logistic regression results by conducting sensitivity analyses using propensity score methods. We used inverse weighted probabilities, which compare what we would expect if everyone was HIV positive to what we would expect if everyone was HIV negative [11]. We conducted three types of analyses: using data from all participants, restricted analysis to propensity scores within the range of largest propensity score among HIV negatives and the least propensity score among HIV positives (i.e., common support), and restricted analysis to propensity scores between the 2.5 and 97.5 percentiles. In order to assess potential effect measure modification by other participant characteristics, we computed unadjusted and adjusted odds ratios of the association between HIV status and the hypertension outcome, stratified by relevant covariates (e.g., quartiles) of BMI. To evaluate associations of HIV status with continuous systolic and diastolic blood pressure, we fitted unadjusted and adjusted linear regression models. Finally, we conducted subgroup analyses among only HIV positive individuals to examine whether CD4 count, viral load and other HIV-related characteristics were associated with systolic and diastolic blood pressure. Analyses were performed using STATA 14 (Stata Corp, College Station, TX, USA).

### Ethics

The procedures for all the parent studies were approved by Makerere University School of Medicine Research Ethics Committee (SOMREC) and Uganda National Council for Science and Technology (UNCST). Administrative authorization was provided by the Nakaseke district health officer as well as the respective hospital and clinic heads. Informed consent was obtained from all eligible participants who signed an informed consent form or thumb-printed and signed by a witness for those who could not read and write.

### Patient and public involvement

This research was done without patient involvement. Patients did not participate in the design of methods and interpretation of results. We did not invite patients to participate in the interpretation of results or to contribute to the writing of this paper for readability and accuracy.

## Results

### Participant characteristics

The final sample consisted of 600 HIV negative and 721 HIV positive individuals (Figure 1). The median age among HIV positive participants in the study was 46.9 years (IQR: 41.5, 53.5) and 44.3 years (IQR: 38.9, 54.3) among HIV negative individuals. Approximately 59.8% of participants living with HIV were female, compared to 55.0% among HIV negative participants. Median BMI for HIV positive participants was 21.1 (IQR: 19.3, 23.6) kg/m^2^ compared to 23.4 (IQR: 21.0, 26.6) kg/m^2^ for HIV negative participants (p < 0.001) (Table 1).

**Table 1:** Demographic and clinical characteristics of participants according to HIV status.

	HIV negative (n = 600)	HIV positive (n = 721)

Socio-demographics		
Age (IQR)	44.3 (38.9, 54.3)	46.9 (41.5, 53.5)
Sex: Female, %	330 (55.0%)	431 (59.8%)
*Marital status, %*		
Single	80 (13.3%)	97 (13.5%)
Married	406 (67.7%)	308 (42.7%)
Cohabiting	25 (4.1%)	156 (21.6%)
Separated/Divorced	39 (6.5%)	63 (8.7%)
Widow/Widower	50 (8.3%)	97 (13.5%)
*Highest level of education, %*		
None/incomplete primary	371 (61.8%)	280 (38.8%)
Primary/incomplete secondary	185 (30.8%)	350 (48.5%)
Secondary or higher	44 (7.4%)	91 (12.7%)
*Fuel source, n (%)*		
Charcoal	54 (9.0%)	83 (11.5%)
Wood	545 (90.8%)	638 (88.5%)
***Behavioral and clinical characteristics***		
Smoking history		
Daily smoker: n (%)	44 (7.3%)	39 (5.4%)
Occasional smoker: n (%)	41 (6.8%)	74 (10.3%)
Non-smoker: n (%)	515 (85.8%)	608 (84.2%)
SBP in mmHg (IQR)	125 (114, 139)	121 (111, 133)
DBP in mmHg (IQR)	80 (73, 88)	76 (69, 85)
Elevated BP: n (%)	361 (60.2%)	345 (47.8%)
Hypertension: n (%)	201 (33.5%)	173 (23.9%)
BMI in kg/m^2^ (IQR)	23.4 (21.0, 26.6)	21.1 (19.3, 23.6)

Values are shown as median ± IQR unless otherwise indicated. BMI, body mass index; DBP: Diastolic Blood Pressure; SBP: Systolic Blood Pressure. Elevated blood pressure was defined as SBP ≥130 mmHg or DBP ≥80mmHg. Hypertension was defined as SBP ≥140 mmHg and/or DBP ≥ 90mmHg. IQR: Interquartile Range.

Most participants were non-smokers. HIV was well controlled within the sample, with >90% of HIV positive participants on ART (Table 2). Of 485 HIV positive participants for whom viral load information was available, 92.9% had undetectable viral load. Furthermore, among 473 HIV positive individuals for whom CD4 information was available, median CD4 was 478 cells/mm^3^ (IQR: 346 cells/mm^3^, 663 cells/mm^3^).

**Table 2:** Characteristics among HIV positive participants (n = 721).

Characteristics	n (%)

**ART**	
Currently on ART	652 (90.4%)
Not on ART	69 (9.6%)
**ART Duration** (years)	
<1	74 (10.2%)
1–4	300 (41.6%)
5–9	196 (27.2%)
10+	77 (10.8%)
*Missing*	74 (10.2%)
**Viral Load (VL)**	
Undetectable VL	451 (62.6%)
Detectable VL	34 (4.7%)
*Missing*	236 (32.7%)
**CD4+T cells (cells/mm^3^)**	
<200	39 (5.4%)
200–499	212 (29.4%)
≥500	222 (30.8%)
*Missing*	248 (34.4%)

ART: Anti-retroviral therapy, CD4+T: cluster of differentiation CD4 lymphocytes.

### Prevalence of hypertension and distribution of blood pressure by HIV status

**Figure 2:**
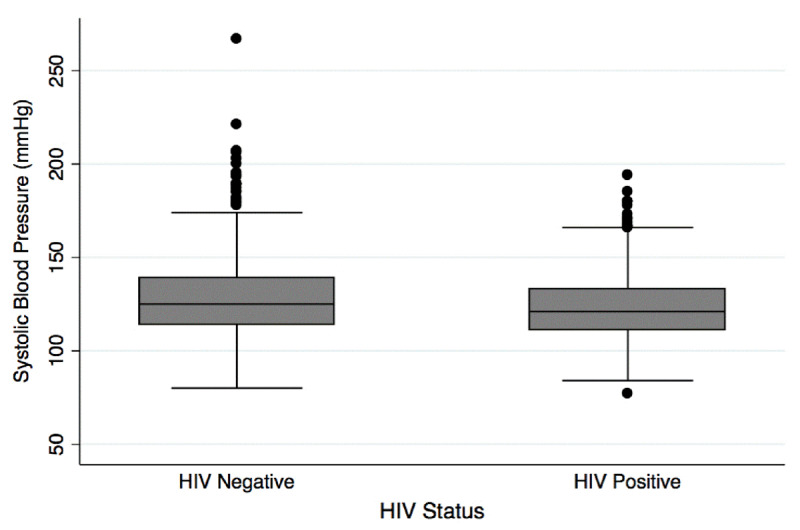
Distribution of systolic blood pressure by HIV status.

**Figure 3:**
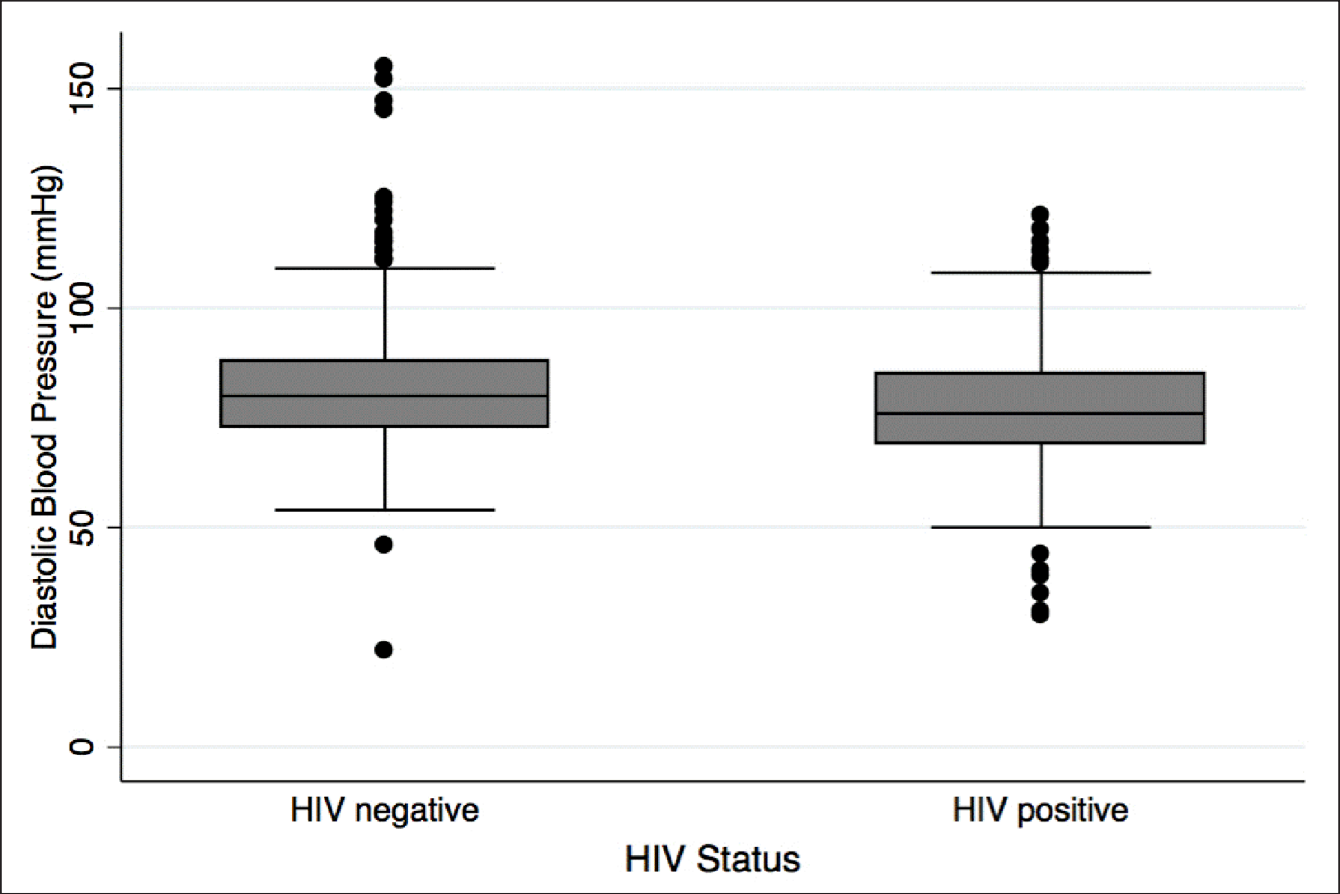
Distribution of diastolic blood pressure by HIV status.

The prevalence of hypertension was 33.5% among HIV negative participants and 23.9% among HIV positive participants (Table 1). The prevalence of elevated blood pressure, defined as systolic blood pressure ≥130 mmHg or diastolic blood pressure ≥80 mmHg, was 60.2% among HIV negative participants and 47.8% among HIV positive participants (Table 1). As shown in box plots, median systolic blood pressure (Figure 2) and median diastolic blood pressure (Figure 3) were higher among HIV negative participants when compared to those that were HIV positive.

### Association between HIV and hypertension

In Table 3, we show the unadjusted and adjusted odds ratios of the association between HIV status and hypertension. Adjusted results showed lower odds of hypertension among participants living with HIV compared to HIV negative individuals (adjusted OR = 0.66, 95% CI 0.50 to 0.88). Continuous age was associated with higher odds of hypertension (adjusted OR = 1.05, 95% CI 1.03 to 1.06), as was continuous BMI (adjusted OR = 1.08 95% CI 1.05, 1.12). Adjusted odds ratios for associations with sex, marital status, biomass use (charcoal/other vs. wood) and household size were not statistically significant. Current smoking status was not significantly associated with hypertension; however, the direction was counterintuitive, with those who reported being occasional or daily smokers having lower odds of hypertension compared to non-smokers.

**Table 3:** Logistic regression results: Associations of HIV status, socio-demographic, and behavioral characteristics with hypertension.

	Unadjusted OR, 95% CI	Adjusted OR, 95% CI

HIV Status: positive vs. negative	0.63 (0.49, 0.80)***	0.66 (0.50, 0.88)**
***Socio-demographics***		
Sex: Female vs. Male	0.96 (0.75, 1.23)	0.80 (0.60, 1.08)
Age	1.04 (1.02, 1.05)***	1.05 (1.03, 1.06)***
*Marital status*		
Single	Ref	Ref
Married	1.08 (0.75, 1.57)	1.15 (0.76, 1.73)
Cohabiting	0.94 (0.59, 1.51)	1.32 (0.80, 2.18)
Separated/Divorced	1.07 (0.62, 1.84)	1.05 (0.60, 1.86)
Widow/Widower	1.19 (0.73, 1.92)	1.04 (0.62, 1.74)
*Highest level of education*		
None/incomplete primary	Ref	Ref
Primary/incomplete secondary	1.10 (0.85, 1.41)	1.29 (0.98, 1.71)
Secondary or higher	1.03 (0.68, 1.56)	1.19 (0.76, 1.87)
Biomass use: Charcoal/Other vs. Wood	1.12 (0.75, 1.68)	1.02 (0.66, 1.58)
Household size	0.99 (0.95, 1.04)	0.97 (0.92, 1.02)
***Behavioral and clinical characteristics***		
Current smoker		
Non-smoker	Ref	Ref
Occasional smoker	0.90 (0.58, 1.38)	0.83 (0.52, 1.31)
Daily smoker	0.58 (0.33, 1.01)	0.56 (0.31, 1.02)
Body Mass Index *(kg/m^2^)*: Median (IQR)	1.07 (1.04, 1.09)***	1.08 (1.05, 1.12)***

IQR: Interquartile Range; OR: Odds ratio. Adjusted models included all variables within the table. P-value: statistical significance (0.049–0.01)*; 0.009–0.001**; less than 0.001***.

Results of propensity score analyses for the association between HIV status and hypertension were in the same direction and of similar magnitude as above analyses (i.e., lower odds of hypertension among HIV positive participants). We obtained the following results: using all propensity scores (adjusted OR = 0.73, 95% CI 0.55, 0.97; p = 0.033), after restriction to common support (adjusted OR = 0.73, 95% CI 0.55, 0.98; p = 0.037), and after trimming at the fifth percentiles (adjusted OR = 0.70, 95% CI 0.52 to 0.94; p = 0.019). We also calculated odds ratios for the association of HIV with hypertension stratified by relevant covariates (Table 4). Overall, findings were consistent with lower odds of hypertension among participants living with HIV compared to participants who were HIV negative. Of note, among BMI quartiles, the lower odds of hypertension among HIV positive participants compared to those who were HIV negative was statistically significant and strongest in the lowest and highest quartiles of BMI.

**Table 4:** Logistic regression results showing associations of HIV status (positive vs. negative) with binary hypertension variable, after stratification by participant characteristics.

	Unadjusted OR, 95% CI	Adjusted OR, 95% CI

**Age**		
Age < 55 years	0.59 (0.45, 0.79)***	0.61 (0.44, 0.86)**
Age ≥ 55 years	0.77 (0.48, 1.24)	0.86 (0.46, 1.59)
**Sex**		
Male	0.85 (0.60, 1.24)	0.78 (0.51, 1.20)
Female	0.49 (0.36, 0.68)***	0.55 (0.38, 0.84)**
**Highest level of education**		
None/incomplete primary	0.68 (0.48, 0.97)*	0.78 (051, 1.19)
Primary/incomplete secondary	0.50 (0.34, 0.74)***	0.56 (0.36, 0.88)*
Secondary or higher	0.65 (0.30, 1.43)	0.61 (0.23, 1.59)
**Body Mass Index (in kg/m^2^)**		
Quartile 1: ≤20.01	0.69 (0.38, 1.24)	0.44 (0.21, 0.93)*
Quartile 2: 20.02–22.05	1.17 (0.71, 1.92)	0.93 (0.52, 1.65)
Quartile 3: 22.06–24.95	0.70 (0.43, 1.12)	0.66 (0.38, 1.14)
Quartile 4: ≥24.96	0.47 (0.29, 0.77)**	0.41 (0.22, 0.75)**
**Smoking status**		
Non-smoker	0.62 (0.48, 0.80)***	0.62 (0.45, 0.84)**
Occasional smoker	0.58 (0.25, 1.34)	0.84 (0.27, 2.59)
Daily smoker	0.85 (0.28, 2.55)	1.40 (0.25, 7.75)
**Household size**		
<6 members	0.60 (0.44, 0.80)***	0.72 (0.51, 1.00)
≥6 members	0.66 (0.43, 1.03)	0.51 (0.30, 0.89)*
**Biomass use**		
Charcoal/other	0.55 (0.26, 1.20)	0.42 (0.15, 1.21)
Firewood	0.63 (0.49, 0.82)***	0.65 (0.49, 0.89)**

OR: Odds Ratio. Adjusted models included all variables within the table. P-value: statistical significance (0.049–0.01)*; 0.009–0.001**; less than 0.001***.

### Association between HIV and blood pressure

We summarize single and multivariable linear regression of the association between HIV and blood pressure in Table 5. After adjustment for relevant confounders, we observed lower systolic blood pressure (adjusted difference: –5.07 (95% CI –7.40 to –2.37, p < 0.001) and diastolic blood pressure (adjusted difference: –4.02 (95% CI –5.57, –2.49, p < 0.001) among HIV positive participants compared to HIV negative participants. BMI was associated with higher systolic blood pressure (adjusted difference: 0.88; 95% CI 0.62, 1.13; p < 0.001) and diastolic blood pressure (adjusted difference: 0.61; 95% CI 0.44, 0.77; p < 0.001).

**Table 5:** Linear regression results: Associations of HIV status, socio-demographic and behavioral characteristics with continuous systolic and diastolic blood pressure.

	Systolic Blood Pressure		Diastolic Blood Pressure	

	Unadjusted difference (95% CI)	Adjusted difference (95% CI)	Unadjusted difference (95% CI)	Adjusted difference (95% CI)

**HIV Status: positive vs. negative**	–5.85 (–7.97, –3.72)***	–5.07 (–7.40, –2. 37)***	–4.46 (–5.983, –3.09)***	–4.02 (–5.57, –2.49)***
**Socio-demographics**				
**Sex: Female vs. Male**	0.49 (–1.67, 2.66)	–1.93 (–4.36, 0.49)	–0.17 (–1.57, 1.23)	–1.60 (–3.19, –0.00)*
**Age**	0.49 (0.38, 0.60)***	0.54 (0.43, 0.66)***	0.09 (0.02, 0.16)*	0.13 (0.06, 0.21)**
**Marital status**				
**Single**	Ref	Ref	Ref	Ref
**Married**	–1.06 (–4.32, 2.21)	–0.37 (–3.64, 2.90)	0.03 (–2.08, 2.14)	0.25 (–1.90, 2.40)
**Cohabiting**	–1.57 (–5.67, 2.54)	1.72 (–2.31, 5.74)	–1.34 (–4.00, 1.31)	0.94 (–1.71, 3.59)
**Separated/Divorced**	–0.21 (–5.04, 4.62)	–0.40 (–4.99, 4.19)	0.45 (–2.67, 3.58)	0.81 (–2.21, 3.83)
**Widow/Widower**	3.71 (–0.63, 8.04)	1.83 (–2.39, 6.04)	0.53 (–2.27, 3.34)	1.14 (–1.63, 3.91)
**Highest level of education**				
**None/incomplete primary**	Ref	Ref	Ref	Ref
**Primary/incomplete secondary**	–1.11 (–3.38, 1.17)	0.96 (–1.32, 3.24)	1.16 (–0.31, 2.62)	2.42 (0.92, 3.92)**
**Secondary or higher**	2.13 (–5.81, 1.56)	0.12 (–3.55, 3.78)	–0.91 (–3.28, 1.47)	0.36 (–2.05, 2.77)
**Biomass use: Charcoal/Other vs. Wood**	0.99 (–2.52, 4.50)	–0.55 (–4.00, 2.89)	–0.28 (–2.54, 1.99)	0.02 (–2.24, 2.29)
**Household size**	–0.21 (–0.58, 0.16)	–0.34 (–0.73, 0.04)	–0.13 (–0.38 0.11)	–0.32 (–0.57, –0.06)*
**Behavioral and clinical characteristics**				
**Current smoker**				
**Non-smoker**	Ref	Ref	Ref	Ref
**Occasional smoker**	–0.59 (–4.40, 3.22)	–1.53 (–5.30, 2.23)	–0.49 (–2.95, 1.97)	–0.26 (–2.73, 2.22)
**Daily smoker**	–2.77 (–7.20, 1.66)	–3.35 (–7.76, 1.06)	–1.72 (–4.58, 1.14)	–1.25 (–4.15, 1.64)
Continuous **BMI *(kg/m2)***	0.80 (0.56, 1.03)***	0.88 (0.62, 1.13)***	0.61 (0.46, 0.76)***	0.61 (0.44, 0.77)***

BMI: Body mass index presented as median and Interquartile range. Adjusted models included all variables within the table. P-value: statistical significance (0.049–0.01)*; 0.009–0.001**; less than 0.001***.

Compared to those with none/incomplete primary education, those who completed primary school/had some secondary education had higher diastolic blood pressure (adjusted difference: 2.42, 95% CI 0.92 to 3.92), while no significant difference was observed among those who completed secondary education (adjusted difference: 0.02; 95% CI –2.24, 2.29).

### Subgroup analysis among HIV positive participants

Subgroup analysis among HIV positive participants with information on CD4 and viral load are shown in Table 6. We did not observe statistically significant associations of CD4 < 300 cells/mm^3^, detectable viral load or reporting being on ART on continuous systolic or diastolic blood pressure. Furthermore, after adjustment, we did not observe statistically significant associations of hypertension (defined as SBP ≥140 mmHg/DBP ≥90mmHg) with detectable viral load compared to undetectable viral load (aOR = –1.83; 95% CI –7.95, 4.29) or not taking ART versus taking ART (aOR = –2.34, 95% CI –6.60, 1.93).

**Table 6:** Linear regression results: Subgroup analysis showing associations of HIV-related characteristics with continuous systolic and diastolic blood pressure.

	Systolic Blood Pressure		Diastolic Blood Pressure	

	Unadjusted difference (95% CI)	Adjusted difference (95% CI)	Unadjusted difference (95% CI)	Adjusted difference (95% CI)

**CD4 T cell count**				
≥300 cells/mm^3^	Ref	Ref	Ref	Ref
<300 cells/mm^3^	–0.55 (–4.75, 3.64)	–1.50 (–5.56, 2.56)	1.79 (–2.49, 6.08)	–0.79 (–3.60, 2.02)
**Viral load**				
Undetectable	Ref	Ref	Ref	Ref
Detectable	–0.67 (–7.07, 5.73)	–1.83 (–7.95, 4.29)	–0.29 (–3.18, 2.59)	1.68 (–2.53, 5.90)
**ART treatment**				
Currently taking	Ref	Ref	Ref	Ref
Not taking	–3.11 (–7.55, 1.32)	–2.34 (–6.60, 1.93)	0.40 (–2.61, 3.41)	0.75 (–2.21, 3.72)
**ART duration**				
<5 years	Ref	Ref	Ref	Ref
5–9	–1.54 (–4.68, 1.59)	–1.66 (–4.70, 1.37)	–1.09 (–3.20, 1.03)	–0.84 (–2.94, 1.25)
10+	–1.73 (–6.18, 2.71)	–3.15 (–7.49, 1.19)	–0.99 (–3.99, 2.01)	–1.35 (–4.34, 1.65)

ART: Anti-retroviral therapy, CD4+T: cluster of differentiation CD4 lymphocytes. Adjusted analyses included age, marital status, highest level of education, biomass use, household size and current smoking status. P-value: statistical significance (0.049–0.01)*; 0.009–0.001**; less than 0.001***.

## Discussion

In this study, we observed high prevalence of hypertension among PLHIV attending ambulatory ART clinics and HIV negative individuals living within the same rural district of Uganda. HIV was well-controlled within the study sample, with over 90% of HIV positive participants being on ART. The prevalence of hypertension was 23.9% among HIV positive participants and 33.5% among HIV negative participants and were similar to those reported in a recent study in Senegal [2] but lower than the global prevalence estimated via a meta-analysis that included studies from America, Europe, Africa and Asia (23.9% vs 34.7%) [12]. Being HIV positive was associated with lower odds of hypertension even after adjusting for traditional risk factors of hypertension (e.g., age, BMI and smoking) consistent with other studies in Uganda [6,13]. For example, a previous large population-based study in Western and Eastern communities of Uganda showed that HIV negative individuals had 20% higher odds of hypertension compared to HIV positive individuals [7]. Another study from West Africa found a hypertension prevalence of 22% among PLHIV and 32% among HIV negative individuals in 2015. Other studies assessing the association between HIV status and hypertension have found mixed results, with some studies suggesting a lack of association [14] and others reporting increased risk of hypertension on ART [12]. In our study, we only examined data for adults aged 35 years and above, which might also explain the slightly higher prevalence of hypertension than that reported in the Ugandan population studies that recruited slightly younger participants (18 years and above) [4,6,7]. The overall prevalence reported in these studies is 14% [7], 15% [6] and 26.4% [4].

The mechanisms that explain our findings are not fully understood, but there are possible explanations for the lower blood pressure observed in PLHIV who attend ART clinics compared to HIV negative individuals living in the same community.

First, the lower odds of high blood pressure among PLHIV than in HIV negative individuals could be due to differences in immunological profiles. CD4 T cells are critical in driving hypertension among HIV negative individuals and there is indirect evidence from early studies that T lymphocytes in target organs drive inflammation and hypertension [15]. Moreover, it is suggested that hypertension among PLHIV is a phenomenon of immune suppression and reconstitution [16]. Initiating ART at lower baseline CD4 counts intensifies the risk of immune suppression-reconstitution [17] and leads to increases in blood pressure or vascular changes among PLHIV [18]. A majority of the PLHIV included in our studies have been on ART since 2010 when guidelines recommended earlier ART initiation: first at CD4 less than 350 cells/mm^3^ (2010) [19], then CD4 less than 500 cells/mm^3^ (2013) [20]. By 2016, the recommendation was initiation of ART irrespective of CD4 count [21]. It is possible that they were initiated on ART at low baseline CD4 count but not low enough to elicit the immune suppression-reconstitution phenomenon. A study in Cameroon showed that participants who had CD4 cell counts greater than 350 cells/μL were three times more likely to have hypertension than those with CD4 cells less than 350 cells/μL, but there was no linear relationship between CD4 cell count and hypertension [22]. Our study was not powered to assess blood pressure at different CD4 thresholds; however, there were no significant differences in BP among persons with CD4+ count greater than or equal to 300 cells/mm^3^ and those with less than 300 cells/mm^3^.

Additional factors that could have influenced our findings include type of ART regimens and ART duration. The ART regimens prescribed for these participants were according to the World Health Organization (WHO) ART guidelines of 2010 plus. These guidelines recommended use of Tenofovir (in place of stavudine), Zidovudine and efavirenz as first line; whereas, the use of protein inhibitors (PIs) were limited to PLHIV who fail on the first line regimen [19,20]. The association of the drugs used in the first line regimens is not clear, but PIs have been implicated in increased hypertension risk [16]. Therefore, the use of relatively benign ART regimens may explain the lack of association between HIV positive status and hypertension in our study population. Findings from a longitudinal study showed that increased ART duration is an independent predictor of hypertension [23]. Moreover, it is said that the longer the duration of ART the higher the risk of high blood pressure, and studies have shown that most times PLHIV become hypertensive after two years of certain ART regimens and systolic blood pressure continues to increase after five years of ART [24]. Our ability to study further the effects of ART duration was limited by the cross-sectional design of the study, but still there was no statistically significant difference in systolic and diastolic blood pressure of PLHIV who had been taking ART for less than five years or more than five years.

Another potential explanation for why blood pressure is somewhat lower among PLHIV than people who are HIV negative is improved awareness about health promoting habits. This is because PLHIV have easy accessibility and usage of health services at their ART clinics. Awareness influences health seeking behaviors for patients with or at risk of hypertension [25]. Moreover, PLHIV who are in care and on ART frequently access different cadres of healthcare workers, including clinicians, counselors and pharmacists, with a frequency of two to three months in most countries in SSA [26]. Thus, the increased interface with the healthcare system provides an opportunity to have their BPs monitored regularly, to receive earlier diagnosis and to initiate preventive interventions, including health education talks on healthy eating and regular exercise [27]. For example, hypertensive respondents in a qualitative study acknowledged that their awareness about having hypertension was dependent on having their BP readings taken either at a health facility or community outreach [25]. Nonetheless, the lower BMI and lower rates of daily smoking (5.4% vs. 7.3%) in our study among PLHIV compared to HIV negative individuals could be attributed to improved lifestyles (e.g., regular exercises), avoiding risky behaviors like smoking and improved diet or use of antihypertensive medication. Also, in a large community-based study, people who were HIV positive and on ART were more likely to be aware of and treated for high blood pressure compared to HIV negative individuals [27].

The observed significant associations in the HIV-hypertension association in both the unadjusted analyses and adjusted analyses lend support to our findings that there exists a relationship between HIV status and hypertension, separate from that which might be mediated by BMI and the other variables included in analysis. In addition, this relationship appears to be most salient among individuals in the lowest and highest BMI quartiles, suggesting potential modification of the HIV-hypertension relationship by low or high adiposity. We note that there were some differences between persons included in our final analytic sample and those excluded (Appendix Table 1). Notably, HIV negative participants included in the final analytical sample tended to be younger and had a higher BMI than community participants who were excluded from the final sample. On the other hand, HIV positive participants included in our final analytic sample tended to be older and had higher BMI than HIV positive participants who were excluded, leading us to speculate that the observed HIV-blood pressure associations may be more conservative than the true associations. Most published evidence on the association between HIV and blood pressure is about the potential mechanisms that explain the increased odds of raised blood pressure among PLHIV compared to HIV negative individuals [16]. The mechanisms explaining lower blood pressure among PLHIV compared to HIV negative individuals seen in the present study and past studies remain unknown and reinforces the need for more studies examining associations between HIV and blood pressure and potential mechanisms underlining these associations.

Our study has several strengths. It was conducted in a large ART treatment center serving villages within a single district in Uganda. The HIV positive cohort was recruited from the HIV clinic—an ambulatory clinic where PLHIV walk in from the surrounding villages for their routine HIV care and support services. The HIV negative comparator group was drawn from within the same district. In addition, our results remained robust even after sensitivity analyses. The interpretation of our results, however, must take into account some potential limitations. First, we compared data collected months apart from an HIV clinic-based sample and a population-based sample. Moreover, our results may be confounded by improved access to care and health seeking behaviors of the HIV positive individuals. Second, the definition of HIV negative status in the population-based sample relied on self-report without confirmatory testing; therefore, it is possible that some individuals who were HIV negative by self-report were actually HIV positive. However, there is widespread HIV testing both at the clinic and community level in Nakaseke, and the prevalence of HIV in our community cohort was 9%, which is consistent with prior estimates of HIV in our study population. Third, BP was measured in two different settings, yet there are documented differences in BP results according to where the measurements were conducted. For HIV negative participants, it was measured at home, while for HIV positive participants, it was done in clinic. Moreover, due to the cross-sectional nature of the studies, we relied on BP readings taken on a single day, rather than that taken over time, which could have increased the chances of overestimating, in the case of white coat hypertension, and/or underestimating in the case of masked hypertension (especially among HIV positive participants). Generalizability of our findings are potentially limited as we examined only individuals ≥ 35 years, and our final analytic sample included individuals with reproducible lung function (and thus, may be healthier than the general population). Finally, there is potential for residual confounding due to lack of accounting for factors such as BP medication use, which were not collected within the parent lung function studies. If BP medication use was substantially higher among HIV positive individuals than among HIV negative individuals, this could drive the associations observed in this analysis.

## Conclusions

In summary, our findings reveal a high burden of high blood pressure, especially among HIV negative individuals, in a rural district of Uganda. Among PLHIV attending regular ART clinics, the association between HIV and high blood pressure appears to be reduced. However, larger prospective studies are needed to investigate plausible biological, virological, immunological and social mechanisms that might explain the lower blood pressure values among PLHIV when compared to HIV negative individuals. Integration of chronic care including HIV and NCD services has potential to optimize screening and management of hypertension and also to improve accessibility to screening among HIV negative individuals who by virtue of their HIV status may interface with a health worker fewer times than HIV positive individuals.

## Additional File

The additional file for this article can be found as follows:

10.5334/gh.858.s1 Appendix Table 1. Comparison between individuals included vs. excluded in the final analysis.

## Data Accessibility Statement

The data underlying the findings of this study are available upon reasonable request from the corresponding author.
