# Supplementary material for: Association between Blood Pressure and HIV Status in Rural Uganda: Results of Cross-Sectional Analysis
Source: Glob Heart. 2021 Feb 10;16(1):12. doi: 10.5334/gh.858 (PMC7880004; doi:10.5334/gh.858)
Supplement: Appendix Table 1. — Comparison between individuals included vs. excluded in the final analysis. [file gh-16-1-858-s1.pdf]

Appendix Table 1. Comparison between individuals included vs. excluded in the final analysis

Characteristics	Included (n = 600)	Excluded (n = 331)
**Community Sample**		
Age, median (IQR)	44.3 (38.9, 54.3)	50.4 (43.1, 60.3)
Sex (female), %	55.0%	54.5%
BMI, kg/m^2, median (IQR)	23.4 (21.0, 26.6)	22.4 (20.3, 25.4)
Self-reported HIV status		
Negative	100%	23.6%
Positive	--	20.6%
Unknown	--	55.8%
**HIV Positive Participants**		
**Characteristics**	**Included (n = 721)**	**Excluded (n =73)**
Age in years, median (IQR)	46.9 (41.5, 53.5)	42.9 (34.9, 53.7)
Sex (female), %	59.8%	54.7%
BMI in kg/m^2, median (IQR)	21.1 (19.3, 23.6)	20.9 (18.6, 22.8)
HIV status		
**Positive**	100%	100%
